# Poly(A) Polymerase and the Nuclear Poly(A) Binding Protein, PABPN1, Coordinate the Splicing and Degradation of a Subset of Human Pre-mRNAs

**DOI:** 10.1128/MCB.00123-15

**Published:** 2015-06-04

**Authors:** Lisa Muniz, Lee Davidson, Steven West

**Affiliations:** a Wellcome Trust Centre for Cell Biology, Edinburgh, United Kingdom; b Department of Molecular Biology and Biotechnology, University of Sheffield, Sheffield, United Kingdom

## Abstract

Most human protein-encoding transcripts contain multiple introns that are removed by splicing. Although splicing catalysis is frequently cotranscriptional, some introns are excised after polyadenylation. Accumulating evidence suggests that delayed splicing has regulatory potential, but the mechanisms are still not well understood. Here we identify a terminal poly(A) tail as being important for a subset of intron excision events that follow cleavage and polyadenylation. In these cases, splicing is promoted by the nuclear poly(A) binding protein, PABPN1, and poly(A) polymerase (PAP). PABPN1 promotes intron excision in the context of 3′-end polyadenylation but not when bound to internal A-tracts. Importantly, the ability of PABPN1 to promote splicing requires its RNA binding and, to a lesser extent, PAP-stimulatory functions. Interestingly, an N-terminal alanine expansion in PABPN1 that is thought to cause oculopharyngeal muscular dystrophy cannot completely rescue the effects of PABPN1 depletion, suggesting that this pathway may have relevance to disease. Finally, inefficient polyadenylation is associated with impaired recruitment of splicing factors to affected introns, which are consequently degraded by the exosome. Our studies uncover a new function for polyadenylation in controlling the expression of a subset of human genes via pre-mRNA splicing.

## INTRODUCTION

The majority of human pre-mRNAs contain introns that are removed by splicing and undergo 3′-end formation by cleavage and polyadenylation (CPA). Splicing is a two-step process requiring 5 small nuclear RNAs (snRNAs) and around 150 proteins (1). A 5′ splice site is first bound by U1 snRNA, and after recognition of the 3′ splice site by U2AF65, the intron branch point is bound by U2 snRNA. Recruitment of U4, U5, and U6 precedes complex rearrangement of the spliceosome, and following the release of U1 and U4, splicing catalysis takes place, leading to exon ligation.

Many splicing factors are recruited to RNA polymerase II (Pol II) and assemble on introns during transcription (2, 3). Following these cotranscriptional commitment steps, multiple observations concur in showing that splicing catalysis is also frequently cotranscriptional (4–9). Nevertheless, some splicing catalysis occurs following polyadenylation, i.e., some introns are present in polyadenylated RNA, from which they are subsequently excised. This is more often the case for introns closer to the poly(A) signal, as may be expected, since there is less time for splicing prior to CPA (5, 7, 8, 10). Several reports indicate that delayed splicing may have regulatory potential. First, alternative splicing decisions are often more posttranscriptional than constitutive splicing events (5, 11). Second, several reports have shown that splicing of transcripts induced during the inflammatory response is delayed relative to CPA (12–14). Third, retained and detained introns modulate the expression of genes in different cells and in response to stimuli, respectively (15, 16). Finally, it was recently found that many posttranscriptional splicing events are inhibited during heat shock, whereas efficient cotranscriptional splicing is less affected (17). Although these data highlight the existence of delayed intron excision, the mechanisms are not yet fully understood.

In human cells, splicing proceeds via an exon definition mechanism involving interactions across exons (18). Removal of the terminal intron requires factors bound to the poly(A) and 3′ splice sites, and mutation of either signal abolishes both splicing and CPA (19–23). Interactions between *trans*-acting factors include the interaction of cleavage and polyadenylation specificity factor (CPSF) with SF3b and that of U2AF65 with both CF1m and poly(A) polymerase (PAP) (24–28). The involvement of PAP in splicing *in vitro* suggests a possible function for polyadenylation in splicing *in vivo*.

Transcripts destined for splicing following polyadenylation possess a poly(A) tail, which is not present during cotranscriptional splicing. Polyadenylation occurs following cleavage at the poly(A) site and is performed by PAP. By itself, PAP is relatively inefficient, but its activity is enhanced by CPSF and the nuclear poly(A) binding protein, PABPN1 (29–35). PABPN1 plays an additional role in the regulation of poly(A) site selection (36). While efficient polyadenylation has an integral mRNA maturation function, inefficient polyadenylation is associated with degradation by the exosome (37–40). Although the exosome plays a general role in the turnover of aberrant RNA, it can function in competition with mRNA maturation in Saccharomyces cerevisiae (41, 42). The relevance of polyadenylation to human health is clear from the number of diseases that are associated with mutations in PABPN1 and with alterations in polyadenylation (43–46). For instance, an N-terminal alanine expansion in PABPN1 leads to oculopharyngeal muscular dystrophy (OPMD) (46, 47).

In the present study, we reveal a stimulatory function for a poly(A) tail in the splicing of transcripts synthesized from chromosomally integrated β-globin and a subset of endogenous genes. This requires PAP, PABPN1, and a terminal poly(A) tail, whereas internal adenine tracts bound by PABPN1 are unable to stimulate splicing. Complementation experiments further delineate that both the RNA binding and PAP-stimulatory functions of PABPN1 are important for intron excision in this context. Interestingly, an N-terminal alanine expansion of PABPN1 is not completely functional in this process. Finally, exosome depletion stabilizes pre-mRNAs whose splicing is sensitive to polyadenylation. Our findings illuminate an important new function of polyadenylation in the splicing and degradation of a subset of human pre-mRNAs.

## MATERIALS AND METHODS

### Primers and siRNAs.

Details of the primers and small interfering RNAs (siRNAs) are provided in the supplemental material.

### Stable cell line construction.

We previously described the pcDNA5 FRT/TO plasmid containing the WT β-globin (βWT) gene (19). The δ ribozyme (RZ) was made by annealing sense and antisense oligonucleotides containing its sequence before their insertion into a vector prepared by PCR amplification of the βWT gene with β-globin cloning vector F and R primers. The poly(A) tail was added by subsequent PCR with primers incorporating the A-stretch. The histone stem-loop was incorporated into the βδ RZ by using tagged PCR primers containing its sequence. The MENβ triplex-encoding sequence was synthesized by Integrated DNA Technologies and inserted directly upstream of the δ RZ. Internal A-tracts were inserted by PCR amplification of the βWT gene with internal A 20, 40, or 60 forward and reverse primers, as appropriate. For rescue of PABPN1 depletion (see Fig. 6), a DNA that coded for the expression of carboxy-terminally Flag-tagged PABPN1 that was RNA interference (RNAi) resistant was synthesized by Genewiz (see Fig. S1 in the supplemental material). This was cloned in place of the β-globin gene in the βWT plasmid using Gibson Assembly (New England BioLabs). Point mutations were made by site-directed mutagenesis. See the supplemental material for all primer sequences.

### Cell culture.

All cells were grown in Dulbecco's modified Eagle's medium (DMEM) supplemented with 10% fetal calf serum. For transient transfection, 3 μg of reporter plasmid was transfected using JetPrime (Polyplus), and assays were performed 24 h later. For RNAi, siRNAs were transfected into 30-mm dishes by using Lipofectamine RNAiMax (Life Technologies) following the manufacturer's guidelines. For PABPN1 RNAi, cells were harvested at 72 h posttransfection. For all other depletions (and for PABPN1 [see Fig. 8]), two successive siRNA transfections were performed. Stable cell lines were generated by transfecting Flp-IN HEK cells with 3 μg of β-globin plasmid and 7 μg pOG44, using JetPrime (Polyplus) transfection reagent, in a 50% confluent 100-mm dish. At 48 h posttransfection, cells were transferred to a tissue culture flask and grown in the presence of hygromycin (100 μg/ml) and blasticidin (10 μg/ml) until resistant cells emerged. Transcription was induced by growth in medium supplemented with 1 μg/ml tetracycline.

### Antibodies.

PAPγ (NBP1-30060; Novus), PAPα (A301-009A; Bethyl), PABPN1 (ab75855; Abcam), β-actin (AM1021B; Abgent), β-globin (sc-21757; Santa Cruz), histone H3 (ab1791; Abcam), and U2AF65 (U4758; Sigma) antibodies were used.

### RNA isolation.

Total RNA was isolated using TRIzol (Life Technologies). Generally, 1 ml of TRIzol was used directly on a 6-well dish of cells following the manufacturer's guidelines.

### UV cross-linking and RNA immunoprecipitation.

Cells were rinsed twice with ice-cold phosphate-buffered saline (PBS), immediately UV irradiated at 400 mJ/cm^2^ (λ = 245 nm) by using a Stratalinker 1800 apparatus (Stratagene), and then scraped into ice-cold PBS. The cell pellet was resuspended in 400 μl of lysis buffer (50 mM Tris-HCl, pH 7.5, 100 mM NaCl, 1% NP-40, 0.1% SDS, 0.5% sodium deoxycholate) supplemented with protease inhibitors, incubated on ice for 10 min, and sonicated for 10 min (30 s on/30 s off; high setting; Bioruptor instrument). Cellular debris was then pelleted at 13,000 × *g* for 10 min at 4°C. The resulting supernatant was treated with DNase for 1 h at 37°C in the presence of an RNase inhibitor and protease inhibitors and then further sonicated for 10 min. The lysates were then added to protein A/G Dynabeads (Invitrogen) preincubated for 2 h with antibodies (2 μg per immunoprecipitation [IP]). No-antibody controls were performed in parallel. Following overnight rotation at 4°C, the beads were washed twice in wash buffer (20 mM Tris-HCl, pH 7.5, 10 mM MgCl_2_, 0.2% Tween 20), twice in high-salt buffer (50 mM Tris-HCl, pH 7.5, 1 M NaCl, 1 mM EDTA, 1% NP-40, 0.1% SDS, 0.5% sodium deoxycholate), twice in wash buffer, and once in proteinase K buffer (50 mM Tris-HCl, pH 7.5, 10 mM MgCl_2_, 0.5% NP-40, 50 mM NaCl). RNA was then eluted from the beads for 1 h at 50°C by using 50 μl elution buffer (1× proteinase K buffer supplemented with 1% SDS and 20 μg proteinase K). RNA was phenol-chloroform extracted and ethanol precipitated. The RNA was then treated with Turbo DNase for 1 h, phenol-chloroform extracted, and ethanol precipitated. Half of the RNA was reverse transcribed, and the other half was used for a parallel, no-reverse-transcriptase control.

### Northern blotting.

In general, 3 μg RNA was used. RNase H treatment involved prior incubation of the RNA with 100 pmol oligonucleotide and RNase H for 1 h at 37°C. After phenol-chloroform extraction and ethanol precipitation, RNAs were separated in a 5% denaturing polyacrylamide gel and electroblotted onto a Hybond-N nylon membrane (Amersham Biosciences). The membrane was probed with a 5′-terminally labeled oligonucleotide complementary to β-globin exon 3 (β-globin ex3 R probe).

### RT-qPCR.

For reverse transcription-quantitative PCR (RT-qPCR), RNA was treated with Turbo DNase (Ambion) before RT using Inprom II (Promega). cDNA was generally diluted to 30 to 50 μl, of which 1 μl was used for real-time PCR in conjunction with Brilliant III SYBR mix (Agilent Technologies) in a Qiagen Rotorgene machine. Parallel reactions were always performed in the absence of reverse transcriptase to establish that the signals obtained were not due to DNA contamination. Comparative quantitation was used to derive the results. Unless otherwise stated, RT was primed with random hexamers (Qiagen) to detect all transcripts.

### Quantitation.

Real-time PCR experiments were quantitated as follows. For each biological replicate, RNA values were first normalized to a reference transcript. The control sample in the set was then given an arbitrary value of 1, and the test samples were quantitated relative to this value. This procedure was repeated for at least three biological replicates per experiment. In the presented graphs, the average fold differences were plotted together with the standard deviations. Student's *t* test was used to establish the significance of any differences.

## RESULTS

### Synthetic cleavage and polyadenylation support splicing and gene expression in humans.

**FIG 1:**
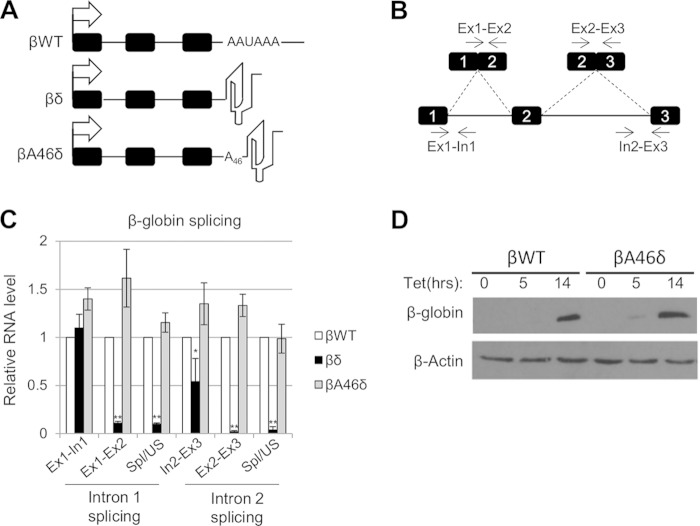
A 3′-templated poly(A) tail supports β-globin splicing. (A) Diagrams of βWT, βδ, and βA46δ genes with 3′ transcript features. Exons are indicated as boxes, and the tetracycline-inducible CMV promoter is shown as an arrow. The following distinguishing features at the 3′ ends of the encoded transcripts are also shown: the poly(A) signal (AAUAAA) for βWT, the δ RZ for βδ, and the A46 template and δ RZ for βA46δ. (B) Diagram illustrating primers and amplicons used for RT-qPCR analysis of β-globin splicing. Exons are numbered. We analyzed splicing efficiency by dividing the signal for spliced RNA by that obtained for the unspliced precursor. (C) Quantitation of RT-qPCR results for analysis of β-globin splicing in cells expressing βWT, βδ, or βA46δ. The level of each RNA species was normalized to the glyceraldehyde-3-phosphate dehydrogenase (GAPDH) mRNA level and is shown relative to that recovered from βWT cells, which was given a value of 1. (D) Western blot analysis of β-globin protein isolated from βWT or βA46δ cells either uninduced or induced with tet for 5 and 14 h, as indicated. β-Actin is shown as a loading control. All error bars represent standard deviations for at least three biological replicates. *, *P* < 0.05; **, *P* < 0.01.

It is well known that splicing and CPA are linked through terminal exon definition, and we previously analyzed the function of individual snRNAs in maintaining this coupling (19). In the present study, we set out to understand how CPA couples 3′-end formation to splicing. We developed a system where CPA could occur in the absence of a poly(A) signal, using the β-globin gene as a reporter. We replaced its poly(A) signal with either a self-cleaving ribozyme (RZ) from hepatitis δ (to create βδ) or the same RZ preceded by 46 adenines (βA46δ) (Fig. 1A). This RZ has been shown to cleave rapidly and efficiently in a variety of contexts in cells (48–50). The native gene (βWT) and these two variants were stably integrated, in single copy, into HEK293 cells. Transcription was driven by a tetracycline (tet)-inducible cytomegalovirus (CMV) promoter.

To test the ability of the modified β-globin genes to support splicing, we performed reverse transcription-quantitative PCR (RT-qPCR) analysis of each cell line after overnight induction with tet. Following cDNA synthesis with random hexamers, qPCR was used to detect unspliced intron 1 (Ex1-In1) and intron 2 (In2-Ex3), as well as spliced exons 1 and 2 (Ex1-Ex2) and spliced exons 2 and 3 (Ex2-Ex3) (Fig. 1B). We also measured the efficiency of splicing by calculating the ratio of spliced to unspliced RNA for both introns. The levels of these species in βδ and βA46δ samples were quantitated relative to those obtained from βWT cells (Fig. 1C). In the case of βδ, some unspliced pre-mRNA was present, albeit at a reduced level for In2-Ex3, but very little spliced mRNA was detected. This shows that 3′-end formation by an RZ instead of a poly(A) site does not support mRNA synthesis, which is concordant with prior studies (48). In contrast, the levels of unspliced and spliced transcripts obtained from βA46δ cells were very similar to those from βWT cells, as was the splicing efficiency. Indeed, Western blotting confirmed that equivalent levels of β-globin protein were produced in the βWT and βA46δ cell lines (Fig. 1D). This is consistent with a previous report demonstrating that synthetic 3′-end formation allows gene expression in budding yeast (51). Importantly, however, we additionally showed that this synthetic CPA supports pre-mRNA splicing in human cells.

### Stabilizing nonpolyadenylated 3′ ends does not promote splicing.

**FIG 2:**
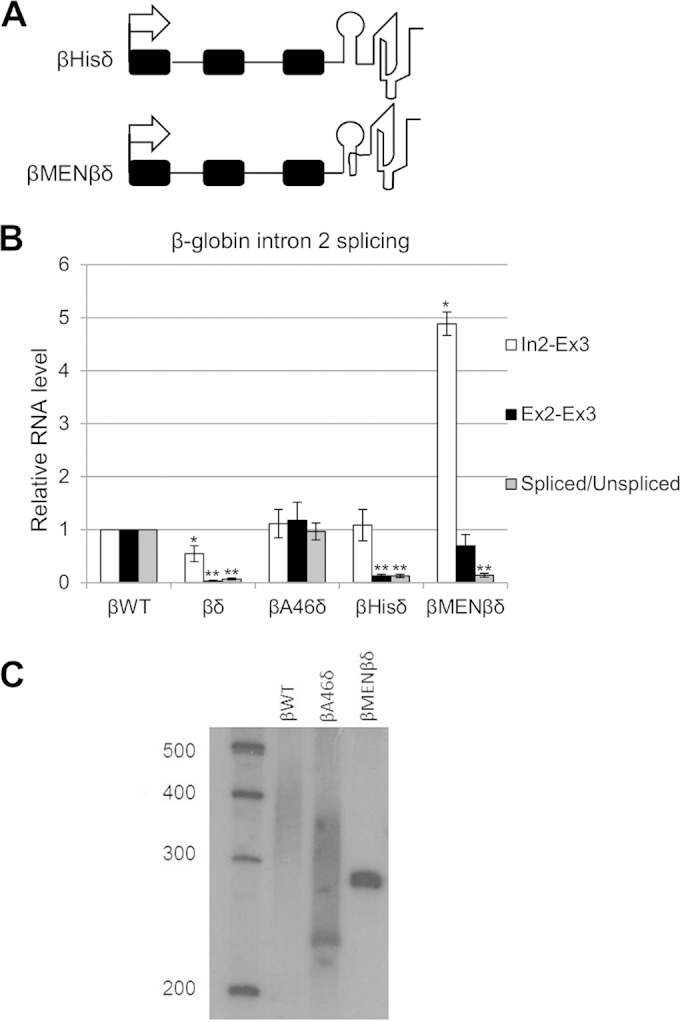
Stable, nonpolyadenylated 3′ ends do not support β-globin splicing. (A) Diagrams of βHisδ and βMENβδ genes integrated into HEK cells. The symbols are the same as in Fig. 1A. The histone stem-loop and MENβ RNA triplex are represented diagrammatically. (B) Quantitation of RT-qPCR results for analysis of β-globin exon 2 and 3 splicing in cells expressing βWT, βδ, βA46δ, βHisδ, or βMENβδ. The level of each RNA species was normalized to the GAPDH mRNA level and is shown relative to that recovered from βWT cells, which was given a value of 1. (C) Northern blot analysis of cells transfected with βWT, βA46δ, and βMENβδ plasmids. RNAs were cleaved by an oligonucleotide targeting β-globin exon 3 before being resolved in the gel. The gel was probed with a 5′-labeled probe targeting exon 3. All error bars represent standard deviations for at least three biological replicates. *, *P* < 0.05; **, *P* < 0.01.

The ability of the templated A-tract to support splicing might be explained by RNA stability effects, since we reproducibly observed smaller amounts of unspliced intron 2 (In2-Ex3) for βδ than for βWT or βA46δ. To test this hypothesis, we generated two more cell lines, in which the 3′ RZ was preceded by a histone stem-loop (βHisδ) or an RNA triplex derived from the noncoding MENβ transcript (βMENβδ) (Fig. 2A). These structures have been shown to stabilize 3′ ends (49, 52). Importantly, the MENβ triplex is predicted to sequester the 3′ end of the RNA within the secondary structure, leaving it unavailable for polyadenylation. We isolated RNAs from βWT, βA46δ, βδ, βHisδ, and βMENβδ cells and analyzed unspliced (In2-Ex3) and spliced (Ex2-E3) β-globin intron 2, together with the splicing efficiency (Fig. 2B). As before, splicing levels of βWT and βA46δ were essentially equivalent, and splicing of βδ RNA was poor, with reduced levels of In2-Ex3 transcripts again seen. The presence of the histone stem-loop upstream of the RZ restored In2-Ex3 levels to normal, but there was still very little spliced RNA present, indicating that simply stabilizing the 3′ end was insufficient to support intron removal. This was also evident for βMENβδ cells, in which pre-mRNA was stabilized 4-fold compared to that in βWT cells but splicing efficiency was similar to that in βδ cells.

We wished to ascertain the polyadenylation status of βA46δ transcripts, so we performed Northern blotting on RNAs from βWT and βA46δ cells (Fig. 2C). As a negative control, we also analyzed βMENβδ transcripts, which are predicted to be nonpolyadenylated due to the 3′triple-helical structure. To obtain a high-resolution view of the 3′ polyadenylation status, RNA within β-globin exon 3 was cleaved by using an oligonucleotide and RNase H before gel electrophoresis. βWT transcripts were observed as a smear, consistent with the presence of a poly(A) tail—something that we confirmed by oligo(dT) and RNase H digestion (see Fig. S2A in the supplemental material). Interestingly, βA46δ transcripts also appeared as a smear rather than a distinct band, indicating a further polyadenylation of the RZ-cleaved end that we confirmed by sequencing (see Fig. S2B). Finally, βMENβδ transcripts are not subject to polyadenylation, as they were present as a single, sharp band corresponding to the RZ cleavage site. We concluded that βA46δ transcripts are further polyadenylated, whereas βMENβδ transcripts are not, and that a 3′ poly(A) tail supports β-globin pre-mRNA splicing but other stable nonpolyadenylated termini do not.

### PABPN1 depletion inhibits splicing that follows poly(A) site cleavage.

**FIG 3:**
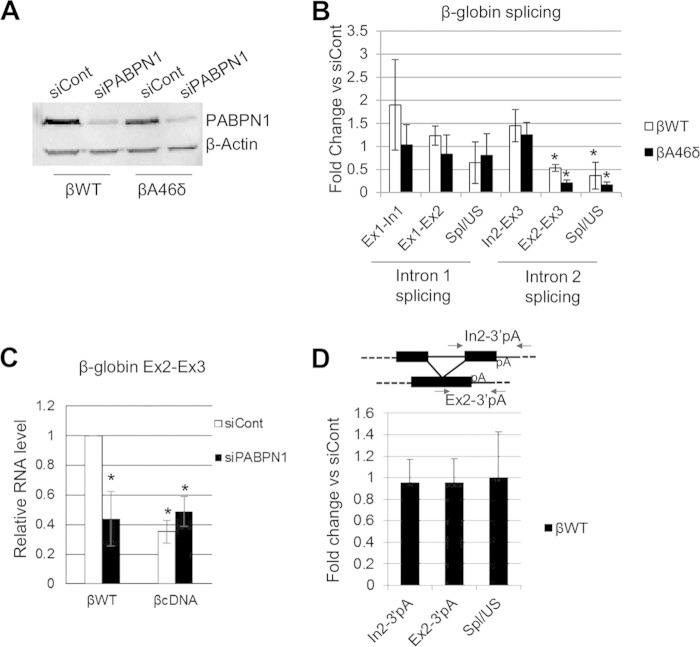
PABPN1 is needed for efficient polyadenylation and splicing of β-globin transcripts. (A) Western blot analysis of PABPN1 in βWT and βA46δ cells treated with control (siCont) or PABPN1-specific (siPABPN1) siRNA. β-Actin is shown as a loading control. (B) Quantitation of RT-qPCR results for analysis of β-globin splicing in βWT and βA46δ cells treated with control or PABPN1-specific siRNA. The level of each RNA species is shown as the fold change compared to the level in control siRNA-treated cells after normalization to GAPDH mRNA. (C) RT-qPCR results for Ex2-Ex3 RNA in stable βWT and βcDNA cells treated with control or PABPN1 siRNA. Values are quantitated relative to those obtained for control siRNA-transfected βWT cells after normalization to GAPDH mRNA. (D) RT-qPCR analysis of cotranscriptional exon 2 and 3 splicing in βWT cells treated with control or PABPN1-specific siRNA. The level of each RNA species is shown as the fold change compared to the level in control siRNA-treated cells after normalization to GAPDH mRNA. The diagram shows the primers used for PCR analysis. All error bars represent standard deviations for at least three biological replicates. *, *P* < 0.05.

We next sought to identify factors recruited to the poly(A) tail that might promote splicing. In humans, nuclear poly(A) tails are bound by PABPN1, which plays an important role in defining the tail length by stimulating and controlling PAP (29–31). To test for a function of PABPN1 in splicing, it was depleted from βWT and βA46δ HEK cells by RNAi (Fig. 3A). RT-qPCR was then used to detect unspliced and spliced intron 1 and intron 2 and to determine their respective splicing efficiencies (Fig. 3B). In both cell lines, PABPN1 depletion caused significant reductions in spliced RNA and splicing efficiency for intron 2. Consistent with this, RNA immunoprecipitation experiments showed that PABPN1 binds to unspliced β-globin transcripts (see Fig. S3 in the supplemental material).

To test the dependence of the PABPN1 effect on the presence of introns, we constructed another cell line (βcDNA) containing intronless β-globin with its native poly(A) site. The βWT and βcDNA cell lines were treated with control or PABPN1 siRNA, and total RNA was isolated. RT-qPCR was then performed to analyze the level of Ex2-Ex3 transcripts (Fig. 3C). Fewer transcripts were produced from the βcDNA gene; however, while PABPN1 depletion reduced the Ex2-Ex3 product in βWT cells, it had no effect in βcDNA cells. This strongly suggests that βWT mRNA is depleted by PABPN1 knockdown, at least in part, due to a pre-mRNA splicing defect. Indeed, a recent report found no effect of PABPN1 on the stability of β-globin mRNA following splicing, although its levels were reduced (53).

Any effect of PABPN1 on splicing is expected to be dependent on a poly(A) tail and therefore requires prior CPA. To test this, we designed primers to detect spliced and unspliced RNAs that were not cleaved at the poly(A) site (Fig. 3D). Next, we performed RT-qPCR by using these primers on samples extracted from control and PABPN1-depleted βWT cells. As described above, we quantitated the levels of spliced and unspliced RNAs, as well as their ratio (as a measure of splicing efficiency). Importantly, PABPN1 did not affect splicing in this assay, consistent with a requirement for a poly(A) tail.

### Internal A-tracts recruit PABPN1 but do not promote splicing.

**FIG 4:**
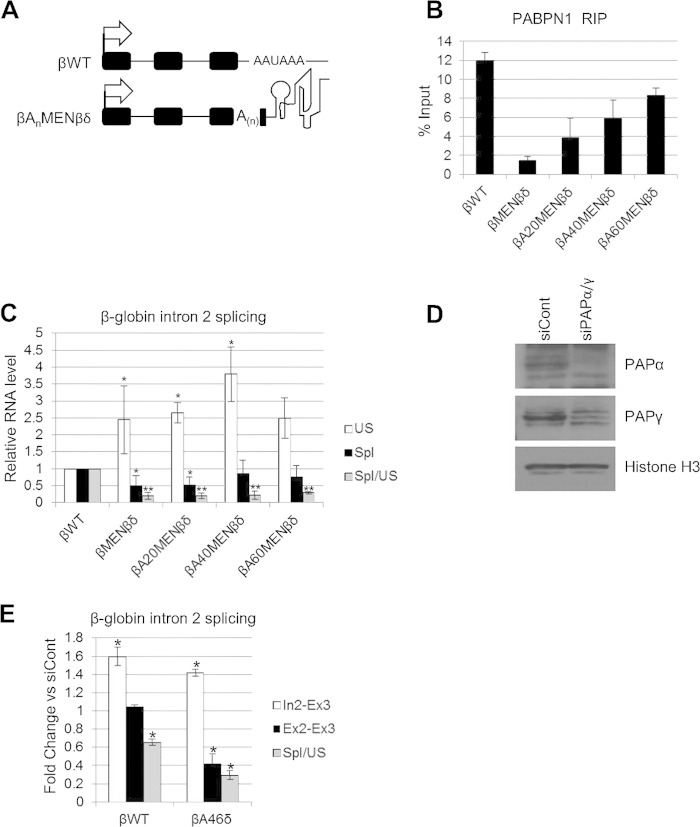
An internal A-tract does not promote splicing in the absence of a 3′-terminal poly(A) tail. (A) Schematics of βWT and βA_n_MENβδ plasmids, with the positions of the internal A-tracts within exon 3 indicated. Other symbols are the same as in Fig. 1A. (B) RNA immunoprecipitation to assay the level of unspliced β-globin RNAs bound by PABPN1 in HeLa cells transiently transfected with the βWT, βMENβδ, βA20MENβδ, βA40MENβδ, or βA60MENβδ construct. Values are expressed as % input following normalization to PSMB3 pre-mRNA levels. (C) RT-qPCR analysis of β-globin exon 2 and 3 splicing in HeLa cells transiently transfected with the βWT, βMENβδ, βA20MENβδ, βA40MENβδ, or βA60MENβδ construct. The level of each RNA species was quantitated relative to that recovered from cells transfected with the βWT construct, which was given a value of 1. (D) Western blot analysis of PAPα and PAPγ proteins in cells treated with control or PAPα- and PAPγ-specific siRNAs. Histone H3 is shown as a loading control. (E) RT-qPCR analysis of exon 2 and 3 splicing in βWT and βA46δ cells treated with control or PAPα- and PAPγ-specific siRNAs. The level of each RNA species is shown as the fold change compared to the level in control siRNA-treated cells after normalization to GAPDH mRNA. All error bars represent standard deviations for at least three biological replicates. *, *P* < 0.05; **, *P* < 0.01. US, unspliced; Spl, spliced.

We next wanted to test whether the effect of PABPN1 was due specifically to its recruitment to the poly(A) tail or related to its function in activating PAP. To do this, we engineered a panel of β-globin plasmids in which 20, 40, or 60 adenines were inserted within β-globin exon 3 (Fig. 4A). This was performed in the context of the βMENβδ construct because the MENβ element prevents further polyadenylation following RZ cleavage (Fig. 2C). Importantly, further Northern analysis confirmed that βA60MENβδ 3′ ends are not subject to polyadenylation (see Fig. S4 in the supplemental material). To test whether PABPN1 could bind the internal A-tracts, we performed RNA immunoprecipitation with cells transfected with the βWT, βMENβδ, βA20MENβδ, βA40MENβδ, and βA60MENβδ plasmids, using the PABPN1 antibody (Fig. 4B). RT-qPCR was used to detect unspliced (In2-Ex3) transcripts, which were bound efficiently by PABPN1 in βWT samples. For comparison, PABPN1 bound very poorly to βMENβδ transcripts, consistent with their lack of a poly(A) tail. Importantly, PABPN1 was effectively recruited to the internal A-tracts, with a binding efficiency correlating positively with the number of A's. This system therefore uncouples 3′-terminal polyadenylation from PABPN1 recruitment, since PABPN1 can bind to internal A-tracts yet the 3′ ends of these MENβδ-based transcripts cannot be polyadenylated.

To check the ability of internally bound PABPN1 to promote splicing, we tested the efficiency of β-globin intron 2 removal in cells transfected with βWT, βMENβδ, βA20MENβδ, βA40MENβδ, and βA60MENβδ by using RT-qPCR (Fig. 4C). Similar to the case in the stable cell lines, βMENβδ transcripts were poorly spliced compared to the βWT transcripts; however, the β-globin splicing efficiency was also low for βA20MENβδ, βA40MENβδ, and βA60MENβδ. This shows that although PABPN1 can bind these internal A-tracts, this binding is insufficient to stimulate splicing following RZ cleavage, consistent with the results of a previous report (54). Thus, simply recruiting PABPN1 to an A-tract does not stimulate intron removal, unless it occurs in the context of a terminal poly(A) tail.

### Poly(A) polymerase is needed for efficient splicing.

We next set out to explain how PABPN1 aids splicing only in the context of a 3′ poly(A) tail. The best-characterized function of PABPN1 bound to poly(A) tails is to stimulate PAP (29–31). An involvement of PABPN1 in stimulating PAP predicts that depletion of polyadenylation activity will also affect splicing. We depleted both PAPα and PAPγ from cells, which is required to reduce polyadenylation activity *in vivo* (53). After confirming successful RNAi (Fig. 4D), βWT and βA46δ cells were treated with control or PAPα/γ siRNA and subjected to RT-qPCR analysis to detect intron 2 (In2-Ex3), spliced exons 2 and 3 (Ex2-Ex3), and splicing efficiency (Fig. 4E). Like PABPN1 depletion, PAP knockdown reduced βWT splicing efficiency, supporting a role for polyadenylation in splicing. Importantly, PAP depletion inhibited βA46δ splicing, suggesting that polyadenylation also contributes to the effect of the terminal poly(A) tail on intron removal following RZ cleavage.

### PAP and PABPN1 are important for a subset of endogenous splicing events.

**FIG 5:**
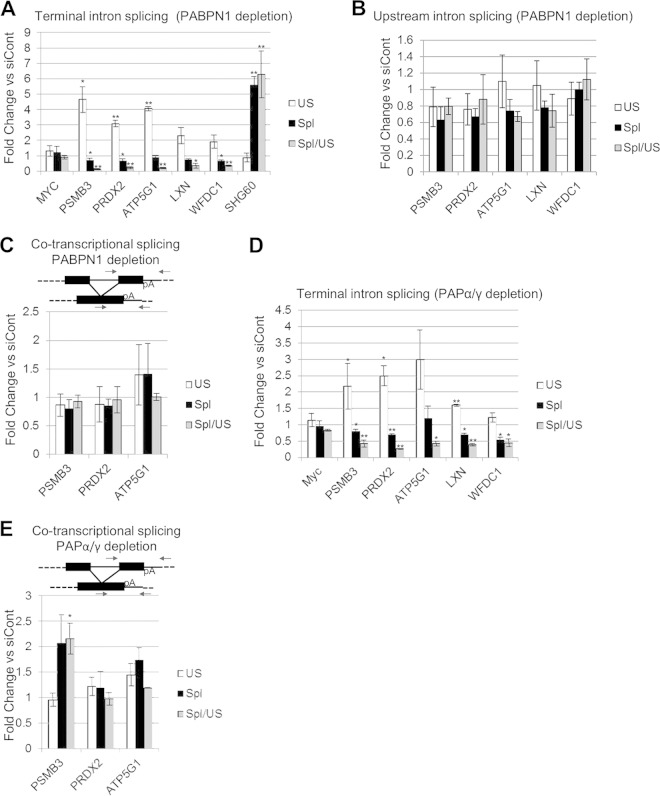
Splicing of a subset of endogenous transcripts requires PABPN1 and PAP. (A) RT-qPCR analysis of Myc, PSMB3, PRDX2, LXN, ATP5G1, and WFDC1 terminal intron splicing, as well as SHG60 noncoding RNA splicing, in PABPN1-depleted cells. The graph shows fold changes in PABPN1-depleted cells relative to control cells after normalization to GAPDH mRNA. (B) RT-qPCR analysis of PSMB3 intron 2, PRDX2 intron 1, LXN intron 1, ATP5G1 intron 2, and WFDC1 intron 1 splicing in PABPN1-depleted cells. The graph shows fold changes in PABPN1-depleted cells relative to control cells after normalization to GAPDH mRNA. (C) RT-qPCR analysis of PSMB3, PRDX2, and ATP5G1 cotranscriptional terminal intron splicing in cells treated with control or PABPN1-specific siRNA. The graph shows fold changes in PABPN1-depleted cells relative to control cells after normalization to GAPDH mRNA. (D) RT-qPCR analysis of Myc, PSMB3, PRDX2, LXN, ATP5G1, and WFDC1 terminal intron splicing in PAPα- and PAPγ-codepleted cells. The graph shows fold changes in PAPα- and PAPγ-codepleted cells relative to control cells after normalization to GAPDH mRNA. (E) RT-qPCR analysis of PSMB3, PRDX2, and ATP5G1 cotranscriptional terminal intron splicing in PAPα- and PAPγ-codepleted cells. The graph shows fold changes in PAPα- and PAPγ-codepleted cells relative to control cells after normalization to GAPDH mRNA. All error bars represent standard deviations for at least three biological replicates. *, *P* < 0.05; **, *P* < 0.01.

A recent study addressed the global impact of PABPN1 depletion on mRNA levels and found that, in most cases, it had little effect (50). However, 227 mRNAs were shown to be reduced >2-fold in this analysis. In light of our findings, we hypothesized that inefficient splicing may explain some of these observations. To test this, five of the downregulated transcripts were selected for further study (PSMB3, PRDX2, LXN, ATP5G1, and WFDC1). The Myc transcript was used as a negative control, as the published transcriptome sequencing (RNA-seq) results revealed no impact of PABPN1 on its expression. As a positive control for PABPN1 effects, we analyzed the SHG60 noncoding RNA, which is degraded in a PABPN1-dependent manner (50). We initially tested terminal intron removal, as we expected it to be most susceptible to a mechanism of splicing occurring after CPA (Fig. 5A). PABPN1 depletion caused an increase in the levels of unspliced PSMB3, PRDX2, LXN, ATP5G1, and WFDC1 transcripts, with a corresponding reduction in splicing efficiency, but no effect was observed in the case of Myc. This pathway is distinct from that controlling SHG60 RNA, as PABPN1 depletion stabilized only spliced transcripts in that case. RNA immunoprecipitation analysis confirmed that PABPN1 bound efficiently to unspliced PSMB3, PRDX2, LXN, ATP5G1, and WFDC1 transcripts (see Fig. S5 in the supplemental material).

Our β-globin data showed that terminal intron splicing was more affected by PABPN1 depletion than proximal intron splicing and that the effect was specific to transcripts that had undergone CPA. Consistent with these findings, PABPN1 depletion had a much smaller effect on the splicing of more promoter-proximal PSMB3, PRDX2, LXN, ATP5G1, and WFDC1 introns (Fig. 5B). Moreover, the splicing of PSMB3, PRDX2, and ATP5G1 transcripts that had not undergone CPA was also unaffected, as measured using a reverse primer beyond the poly(A) site (Fig. 5C). Note that LXN1 and WFDC1 were not analyzed in Fig. 5C, because we were unable to amplify across the final exon to downstream of the poly(A) site by real-time PCR.

Results for β-globin stable cell lines and reporters demonstrated that polyadenylation, rather than PABPN1 itself, mediated the poly(A) tail effect on splicing. Indeed, poly(A) tails were shorter on PRDX2 pre-mRNAs following PABPN1 depletion (see Fig. S6 in the supplemental material). To test this on endogenous transcripts, terminal intron splicing was analyzed for PSMB3, PRDX2, LXN, ATP5G1, and WFDC1 in control or PAPα/γ-depleted cells (Fig. 5D). In line with our β-globin data, PAPα/γ depletion reduced the efficiency of splicing in each case. As expected, this effect required poly(A) site cleavage, as PAPα/γ knockdown did not affect cotranscriptional splicing measured using a reverse primer beyond the PSMB3, PRDX2, and ATP5G1 poly(A) signals (Fig. 5E).

### The RNA binding and PAP-stimulating functions of PABPN1 are important for splicing.

**FIG 6:**
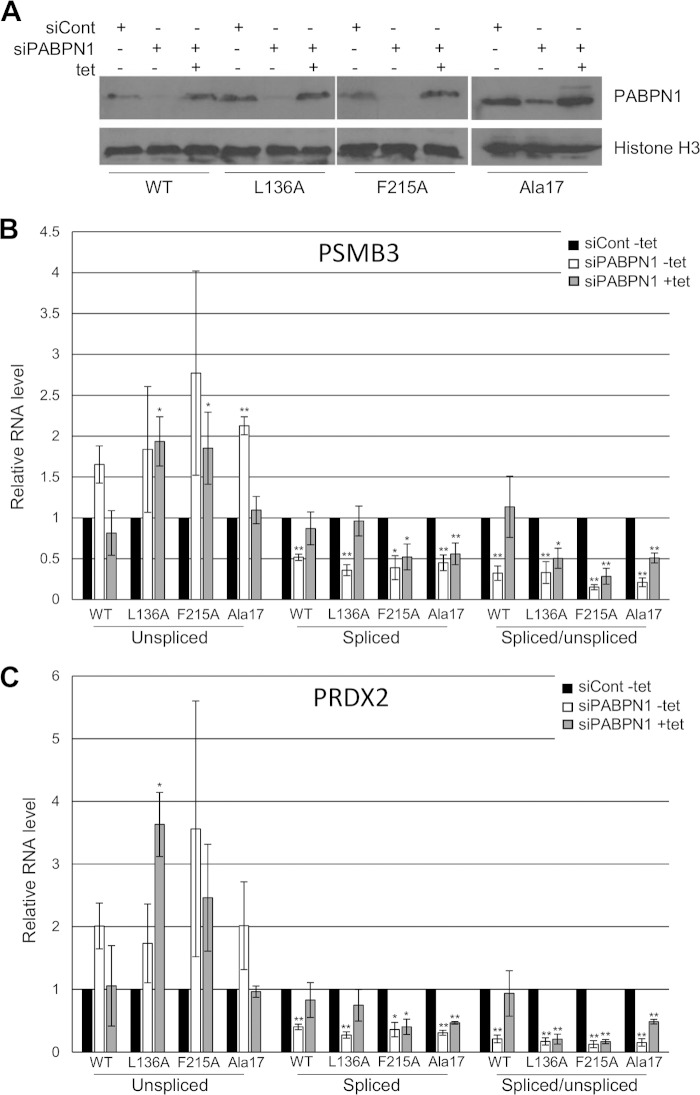
Splicing requires the RNA binding and PAP-stimulating functions of PABPN1. (A) Western blot analysis of PABPN1 in PABPN1 WT, L136A, F215A, and Ala17 cell lines treated with control or PABPN1 siRNA or with PABPN1 siRNA and then tet. The histone H3 protein is shown as a loading control. (B) RT-qPCR analysis of PSMB3 terminal intron splicing in PABPN1 WT, L136A, F215A, and Ala17 cell lines treated with control or PABPN1-specific siRNA or with PABPN1 siRNA and then tet. The levels of spliced and unspliced RNAs are shown, as well as their ratios. In each case, quantitation was performed relative to amounts recovered from cells transfected with control siRNA, which were given a value of 1 after normalization to GAPDH mRNA. (C) The same as panel B, but PRDX2 splicing was analyzed. All error bars represent standard deviations for at least three biological replicates. *, *P* < 0.05; **, *P* < 0.01.

To more robustly test and define the function of polyadenylation in splicing, we developed a system whereby PABPN1 expression could be restored in cells in which the native protein had been depleted by RNAi. We also used this complementation system to test whether key mutants of PABPN1 could support splicing. These were the F215A mutant, which cannot bind RNA (55); the L136A mutant, which cannot stimulate PAP (29); and the Ala17 mutant, which contains an expanded tract of alanine at its N terminus that causes OPMD (46). Stable HEK cell lines were constructed to express RNAi-resistant WT, F215A, L136A, and Ala17 versions of PABPN1 under the control of a tet-inducible CMV promoter. The efficacy of the system was confirmed by Western blotting, which showed that endogenous PABPN1 was depleted by RNAi and that tet induction complemented its loss with the appropriate mutant version (Fig. 6A). Note that tet was added only after PABPN1 was already depleted.

Next, RT-qPCR was used to analyze PSMB3 (Fig. 6B) and PRDX2 (Fig. 6C) splicing in all four cell lines, treated with either control siRNA, PABPN1 siRNA, or PABPN1 siRNA and then tet. In WT cell lines, PABPN1 RNAi increased the level of unspliced RNA, as we had observed before (*P* = 0.055 for PSMB3 and *P* = 0.059 for PRDX2), and reduced the level of spliced transcripts. The effect was not as large as that in Fig. 5, which might have been due to leakage of the tet promoter resulting in some RNAi-resistant PABPN1 expression even in the absence of tet. Crucially, tet addition to PABPN1-depleted cells restored unspliced and spliced RNAs to normal levels.

In L136A cell lines, there was again an increase in pre-mRNA levels and a reduction in spliced RNAs caused by PABPN1 depletion in the absence of tet. When L136A PABPN1 was induced, there was some recovery of spliced PRDX2 and PSMB3 RNAs but not of their unspliced precursors. Thus, splicing efficiency measured by the ratio of spliced to unspliced RNA was not restored to the level seen when WT PABPN1 was induced. Most strikingly, the F215A RNA binding mutation rendered PABPN1 completely unable to restore pre-mRNA and mRNA levels or splicing efficiency to cells depleted of native PABPN1. These experiments provide an insight into the nature of this splicing mechanism. While efficient polyadenylation plays some role, as the L136A mutant does not completely support splicing, the F215A mutation has no restorative effect on splicing as determined by the measures used here. We concluded that PABPN1 binding to 3′-end poly(A) tails underlies its function in splicing and that its ability to stimulate polyadenylation may be required for full efficiency.

Complementation of PABPN1 loss with the expression of Ala17 PABPN1 restored the amounts of unspliced pre-mRNAs to control levels, suggesting that the Ala17 mutant functions normally in the metabolism of these species. However, the Ala17 mutation could not rescue the level of spliced PSMB3 or PRDX2 transcripts, which remained low following induction in PABPN1-depleted cells. Although modest, these effects were statistically significant and potentially reveal a new effect of the OPMD-associated alanine expansion on mRNA biogenesis.

### PABPN1 depletion reduces the cross-linking of splicing factors to terminal introns.

**FIG 7:**
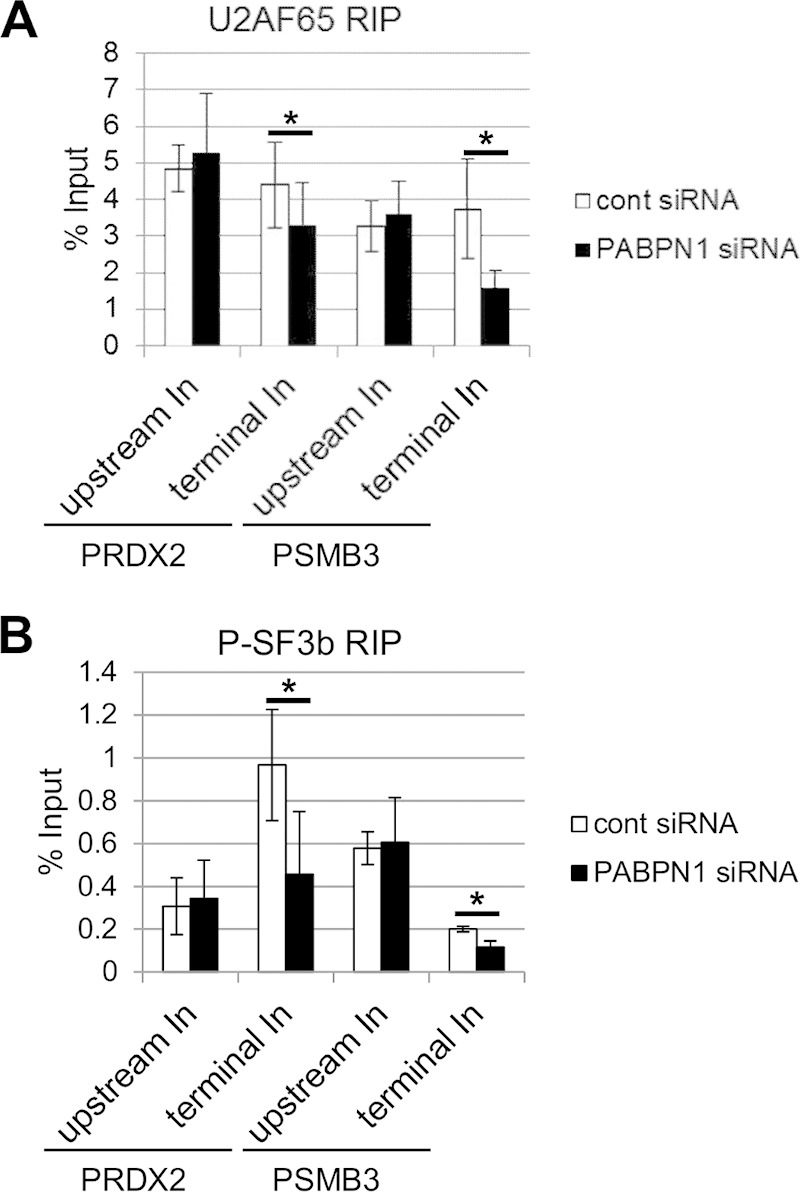
Polyadenylation promotes the binding of splicing factors to affected introns. (A) UV cross-linking followed by immunoprecipitation of RNA from control or PABPN1-depleted cells with a U2AF65 antibody. RT-qPCR was used to analyze upstream or terminal introns from the PRDX2 and PSMB3 genes. The graph shows percentages of input after normalization to Myc In2-Ex3 transcripts. (B) UV cross-linking followed by immunoprecipitation of RNA from control or PABPN1-depleted cells with an antibody to phosphorylated SF3b155. RT-qPCR was used to analyze upstream or terminal introns from the PRDX2 and PSMB3 genes. The graph shows percentages of input after normalization to Myc In2-Ex3 transcripts. All error bars represent standard deviations for at least three biological replicates. *, *P* < 0.05.

PAP has been shown to bind U2AF65 *in vitro*, thereby promoting splicing (28). This suggests that polyadenylation may enhance the recruitment of U2AF65 to affected introns in cells. To test this, we UV-cross-linked control and PABPN1-depleted cells and immunoprecipitated transcripts by using an antibody to U2AF65, which plays a well-established role in terminal exon definition (19, 27, 28). UV cross-linking fixes only direct RNA-protein contacts, enabling us to determine whether the binding of U2AF65 to RNA is affected by PABPN1 depletion. We used RT-qPCR to analyze upstream and terminal introns from the PRDX2 and PSMB3 genes (Fig. 7A). In both cases, a modest but significant reduction in U2AF65 binding to terminal introns was seen following PABPN1 depletion. However, there was little difference in the recovery of upstream introns, which do not depend on PABPN1 for their splicing (Fig. 5). Cross-linking was necessary to stringently observe these differences, which were not apparent in its absence (see Fig. S7 in the supplemental material).

We next performed the same experiment by using an antibody to phosphorylated SF3b155 that specifically detects only activated spliceosomes (Fig. 7B) (56). Similar to U2AF65, less phosphorylated SF3b155 was cross-linked to PSMB3 and PRDX2 terminal introns in PABPN1-depleted cells. As for U2AF65, no difference in cross-linking was observed for upstream introns. These data strongly suggest that PABPN1 and polyadenylation promote the binding of splicing factors to introns. This effect is specific to introns affected by polyadenylation, as those further upstream recruit splicing factors equivalently in control and PABPN1-depleted cells.

### The core exosome and its Rrp6 cofactor target unspliced pre-mRNAs following PABPN1 depletion.

**FIG 8:**
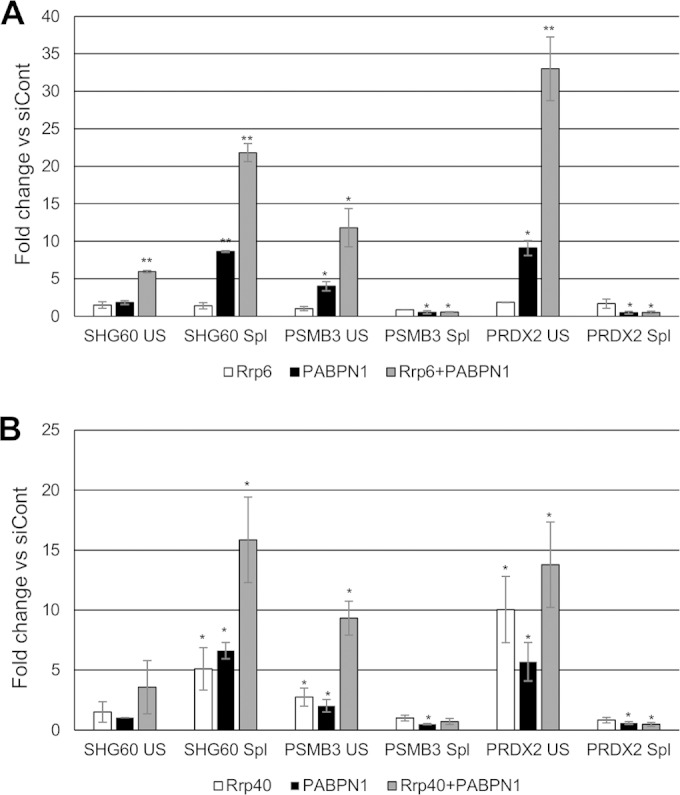
The nuclear and core exosomes degrade PSMB3 and PRDX2 pre-mRNAs. (A) RT-qPCR analysis of control cells and cells depleted of Rrp6, PABPN1, or both Rrp6 and PABPN1. Spliced and unspliced SHG60, PRDX2, and PSMB3 transcripts were analyzed. Values are shown as fold changes relative to the levels in control samples following normalization to GAPDH mRNA. Note that PABPN1 effects are larger than those in previous figures because two rounds of siRNA transfection were performed. (B) RT-qPCR analysis of control cells and cells depleted of Rrp40, PABPN1, or both Rrp40 and PABPN1. Spliced and unspliced SHG60, PRDX2, and PSMB3 transcripts were analyzed. Values are shown as fold changes relative to the levels in control samples following normalization to GAPDH mRNA. Note that PABPN1 effects are larger than those in previous figures because two rounds of siRNA transfection were performed. All error bars represent standard deviations for at least three biological replicates. *, *P* < 0.05; **, *P* < 0.01.

The data described so far show that disruption of the polyadenylation mechanism impairs splicing of a selected set of pre-mRNAs and that this is associated with reduced splicing factor recruitment to affected introns. The RNA exosome complex has been shown to degrade unspliced RNAs and inefficiently polyadenylated transcripts in yeast (37, 42). To test whether this pathway monitors polyadenylation-sensitive splicing events, we depleted cells of the nuclear exosome cofactor, Rrp6. We also analyzed cells depleted of PABPN1 or codepleted of PABPN1 and Rrp6. RT-qPCR was then used to detect unspliced and spliced PSMB3 and PRDX2 transcripts as well as the SHG60 noncoding RNA (Fig. 8A). As expected, PABPN1 depletion resulted in an increase in the level of unspliced RNA, with a corresponding reduction in the recovery of spliced transcripts. Rrp6 depletion alone did not significantly affect the level of unspliced or spliced PRDX2 and PSMB3 RNAs, suggesting that it does not degrade them in the presence of fully efficient polyadenylation. However, there was a striking stabilization of unspliced transcripts from both genes when Rrp6 and PABPN1 were codepleted. This was not accompanied by any substantial change in the levels of spliced transcripts relative to those with PABPN1 single depletion. In the case of SHG60, neither Rrp6 nor PABPN1 depletion affected unspliced RNA levels, but their codepletion resulted in stabilization of this species. These data demonstrate that when PABPN1 is depleted, unspliced precursor transcripts are degraded in an Rrp6-dependent manner.

We next sought to ablate core exosome activity and did so by depleting its Rrp40 component. Again, cells were also treated with PABPN1 siRNAs or codepleted of PABPN1 and Rrp40, and PSMB3 and PRDX2 terminal intron splicing was assayed by RT-qPCR (Fig. 8B). As before, the level of unspliced RNA was increased following depletion of PABPN1, with the spliced transcript level and splicing efficiency being reduced. Unlike for Rrp6, Rrp40 depletion alone stabilized unspliced PSMB3 and PRDX2 transcripts, indicating that they are degraded by the core exosome even in the presence of functional polyadenylation. Like the case for Rrp6 depletion, this was not accompanied by a reduction in the level of spliced RNA. Codepletion of PABPN1 and Rrp40 had a synergistic effect on the stability of PSMB3 pre-mRNA but not PRDX2 pre-mRNA. Rrp40 had an effect similar to that of Rrp6 on SHG60 transcripts, with the exception that its sole depletion stabilized spliced SHG60 RNAs. These data demonstrate that PRDX2 and PSMB3 pre-mRNAs are subject to core exosome-dependent turnover, but their spliced transcripts are not. The lack of an exosome effect on spliced mRNA levels further argues that their reduction following PABPN1 depletion is due to less efficient splicing rather than to degradation.

## DISCUSSION

In this study, we showed that a terminal poly(A) tail promotes the removal of a subset of human introns and that this involves PAP and PABPN1. The removal of polyadenylation-sensitive introns is monitored by the exosome: its core complex targets these pre-mRNAs constitutively, whereas its nuclear cofactor, Rrp6, is only active following PABPN1 knockdown. Interestingly, Ala17 PABPN1 cannot fully restore spliced RNA levels to PABPN1-depleted cells, suggesting that mRNA biogenesis may be affected in OPMD.

Our data demonstrate that the polyadenylation effect is largely specific to terminal intron splicing. This aligns with genome-wide data demonstrating that poly(A) proximal introns are more frequently spliced following polyadenylation (6–8). It is also consistent with established models for exon definition whereby splicing is functionally coupled to CPA (20–22). CPSF is a key factor in this process, and its binding to AAUAAA is controlled by PABPN1 (24, 25, 30). Although our data provide evidence that polyadenylation helps to recruit splicing factors, it is possible that PABPN1 and PAP depletion also prevents CPSF from participating in splicing. PABPN1 has also been shown to suppress poly(A) signals (36), whose activation might disrupt splicing. We find this possibility unlikely, because PAP depletion also inhibits splicing, and under such conditions, PABPN1 would presumably remain able to inhibit poly(A) signals.

The number of splicing events sensitive to polyadenylation is limited based on the fact that very few mRNAs are downregulated following PABPN1 depletion (50). For polyadenylation to affect splicing, poly(A) site cleavage must have occurred before intron excision. An obvious factor that could influence this order of events is having a strong poly(A) signal and relatively weak splice sites in the terminal intron. A visual analysis of sequences around the 3′ splice site of the 5 genes that we studied did not highlight any obvious features that would make them weak. Indeed, the data in Fig. 7 suggest that affected introns recruit U2AF65 and phosphorylated SF3b155 at least as efficiently as unaffected upstream introns. Very strong poly(A) signals may not provide the explanation, because one of our studied transcripts, ATP5G1, has a noncanonical AUUAAA hexamer. It is likely that a combination of factors influences the rate of splicing catalysis versus poly(A) site cleavage and that, when the latter occurs first, polyadenylation increases the efficiency of subsequent intron removal.

In each scenario where we observe reduced splicing, PAP function or recruitment is reduced. For instance, RNAi of PAPα/γ inhibits splicing, and point mutations in PABPN1 that impair its RNA binding or PAP-stimulating functions do not support splicing. Finally, PABPN1 cannot promote splicing when tethered to an internal A-tract in the absence of a terminal polyadenylation reaction. A likely model to explain these data would involve the direct recruitment of splicing factors by PAP, which is tethered to the poly(A) tail most stably in the presence of PABPN1. This is supported by reduced binding of splicing factors to affected introns when PABPN1 is depleted and by the confirmed role of PAP in promoting U2AF65 binding to introns *in vitro* (Fig. 7) (28). Interestingly, biochemical characterization of L136, which is necessary for fully efficient splicing in our experiments, showed that it is required to stimulate polyadenylation but not for PABPN1 to bind RNA and tether PAP (29, 30). Therefore, even in the presence of PAP, efficient polyadenylation may improve the efficiency of splicing, possibly by favoring intron excision over pre-mRNA turnover, thus improving mRNA output.

It is well known that polyadenylation efficiency discriminates stable, functional transcripts from rapidly degraded aberrant RNAs (37, 38). This predicts effects of PABPN1 and PAP depletion on RNA turnover. Indeed, knockdown of either the core exosome (via Rrp40) or its nuclear cofactor, Rrp6, stabilizes both PSMB3 and PRDX2 pre-mRNAs. This effect is evident for Rrp6 only when PABPN1 is codepleted, suggesting that this factor is directed to transcripts only when polyadenylation is impaired. However, Rrp40 depletion stabilized pre-mRNAs even when PABPN1 was still present. This suggests that polyadenylation-sensitive splicing events are in competition with degradation by the core exosome. Interestingly, spliced transcripts showed no substantial exosome effect, indicating that quality control occurs exclusively prior to intron removal. An analogous mechanism was recently uncovered in Schizosaccharomyces pombe, in which the poly(A) binding protein, Pab2, regulates the expression of some spliced transcripts via turnover of their pre-mRNA (57). These data are consistent with a model whereby weak splicing events are monitored by the exosome and, furthermore, where polyadenylation plays a key role in determining whether transcripts are processed or degraded.

There are many ways in which polyadenylation can be regulated to provide scope to change the balance between processing and turnover that we describe. PAP is subjected to a wide range of posttranslational modifications in eukaryotes to regulate its activity. For instance, phosphorylation inhibits PAP during the M phase of the cell cycle, and its nuclear localization stability and association with CPA factors are disrupted by acetylation and sumoylation (58–60). Finally, PARylation of PAP occurs during heat shock, which inhibits polyadenylation (61). Consistent with our data, heat shock also causes widespread inhibition of posttranscriptional splicing, whereas many efficient cotranscriptional splicing events are unaffected (17). Further analyses are required to establish whether regulation of polyadenylation interleaves with splicing and exosome function to modulate gene expression.

Finally, an N-terminal alanine expansion causes OPMD, but the molecular mechanism for the disease is not well characterized (46). Nevertheless, several reports indicate a propensity for the mutant protein to form nuclear aggregates (62). One possible consequence of these aggregates is the depletion of functional PABPN1 from the cell in a dominant negative fashion. In support of this hypothesis, overexpression of the Ala17 mutant recapitulates the effect of PABPN1 knockdown in terms of the effects on poly(A) site selection. Moreover, data in the present study reveal that the Ala17 mutant only partially restores spliced PRDX2 and PSMB3 mRNAs to PABPN1-depleted cells, whereas splicing is fully rescued by the wild-type protein. The possible disease relevance of PABPN1 function in alternative CPA and poly(A) tail-dependent splicing is an interesting topic for further study.

## Supplementary Material

Supplemental material
