# Supplementary material for: Poly(A) Polymerase and the Nuclear Poly(A) Binding Protein, PABPN1, Coordinate the Splicing and Degradation of a Subset of Human Pre-mRNAs
Source: Mol Cell Biol. 2015 Jun 4;35(13):2218–30. doi: 10.1128/MCB.00123-15 (PMC4456446; doi:10.1128/MCB.00123-15)
Supplement: Supplemental material [file supp_35_13_2218__index.html]

Supplemental material 

# Poly(A) polymerase and the nuclear poly(A) binding protein, PABPN1, coordinate the splicing and degradation of a subset of human pre-mRNAs

## Supplemental material

- Supplemental file 1 -

  Fig. S1 (Sequence of synthesized WT PABPN1), S2 [β-Globin RNA and poly(A) tails], S3 and S5 (PABPN1 binding), S4 (Northern blot analysis of β-globin RNA), S6 (ePAT assay), and S7 (Terminal PRDX2 and PSMB3 introns) and Table S1 (Primers)

  PDF, 601K
