# Supplementary material for: Poly(A) Polymerase and the Nuclear Poly(A) Binding Protein, PABPN1, Coordinate the Splicing and Degradation of a Subset of Human Pre-mRNAs
Source: Mol Cell Biol. 2015 Jun 4;35(13):2218–30. doi: 10.1128/MCB.00123-15 (PMC4456446; doi:10.1128/MCB.00123-15)
Supplement: Supplemental material [file MCB.00123-15_zmb999100867so1.pdf]

**Synthesised RNAi resistant PABPN1 (including regions of overlap used for Gibson assembly). Note that the sequence is codon optimised for synthesis purposes**

CCAGCCTCCGGACTCTAGCGTTTAAACTTAAGATGGCCGCTGCAGCTGCAGCAGCTGCTGC
TGCAGGGGCTGCAGGAGGACGAGGCAGCGGGCCAGGTCGGAGACGGCATCTGGTCCCAG
GTGCGGGTGGCGAGGCGGGAGAGGGCGCTCCAGGAGGGGCCGGCGACTACGGTAACGGG
CTGGAATCCGAGGAGCTTGAACCAGAGGAGCTGCTGCTCGAACCTGAGCCCGAACCAGAAC
CAGAGGAGGAACCTCCACGGCCTCGCGCCCCGCCAGGGGCGCCCGGCCCAGGCCCCGGT
AGTGGAGCCCCCGGGTCTCAAGAGGAAGAGGAAGAGCCCGGCCTGGTTGAGGGTGATCCC
GGGGACGGTGCTATCGAAGATCCCGAACTTGAGGCCATCAAAGCAAGAGTGCGAGAGATGG
AAGAGGAAGCAGAGAAACTGAAGGAGCTGCAGAATGAGGTGGAGAAGCAGATGAACATGAG
CCCACCTCCCGGCAACGCCGGCCCTGTGATTATGTCCATCGAGGAGAAGATGGAAGCTGAC
GCTAGATCTATCTATGTGGGTAACGTAGACTACGGTGCGACTGCAGAGGAACTTGAAGCACA
CTTTCACGGATGCGGCAGTGTCAATCGGGTGACTATTCTGTGCGACAAGTTTTCCGGCCATC
CAAAGGGCTTCGCCTATATTGAGTTCTCCGACAAAGAGAGTGTGAGAACTAGCCTGGCATTG
GACGAATCTCTGTTTAGAGGGCGGCAGATTAAAGTGATCCCTAAGAGAACCAACAGACCTGG
CATCTCAACCACAGATCGAGGCTTCCCACGCGCTAGGTATAGAGCGCGCACCACAAATTACA
ACAGCTCTCGCTCTCGGTTTTATAGCGGCTTTAATAGCAGACCGAGGGGACGCGTATATAGA
GGGCGGGCCAGAGCTACTTCCTGGTACTCACCTTACGACTACAAAGACGATGACGACAAGT
AAGCTCGCTTTCTTGCTGTCCAAT

## Supplementary Figure 1

**A**

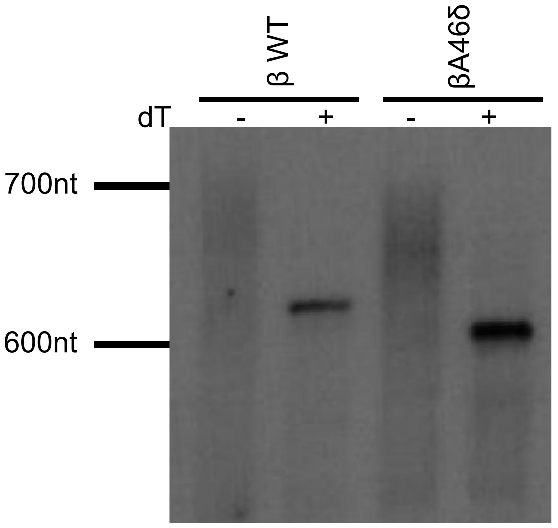


**B**

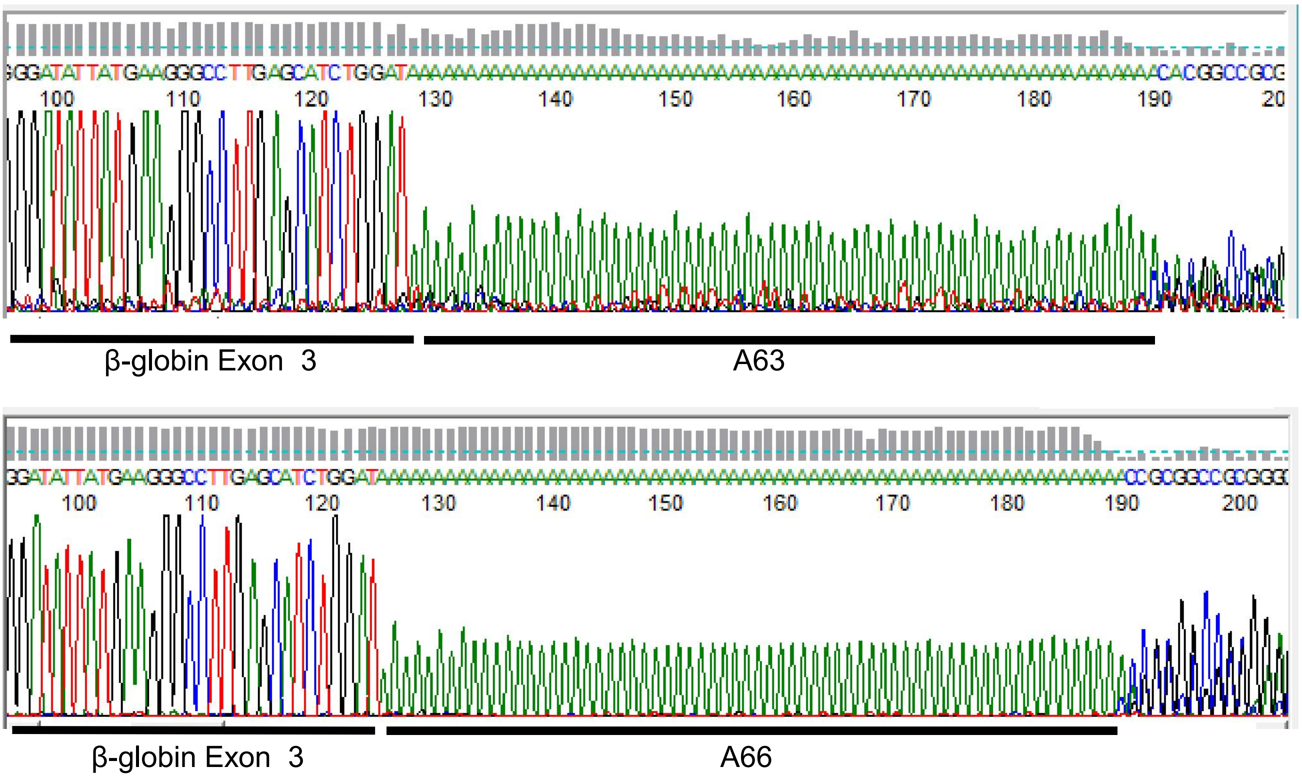


**Supplementary Figure 2**

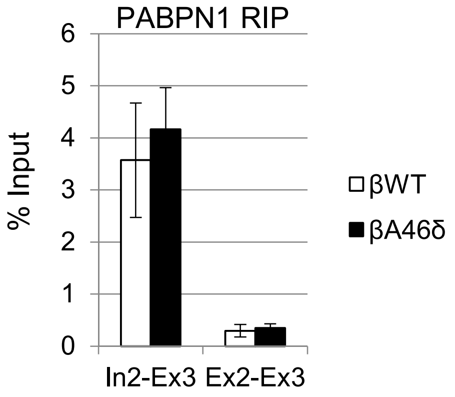


**Supplementary Figure 3**

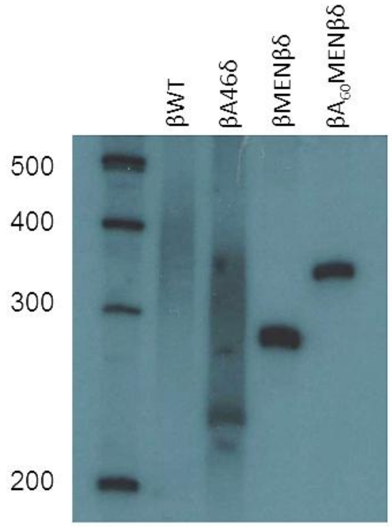


**Supplementary Figure 4**

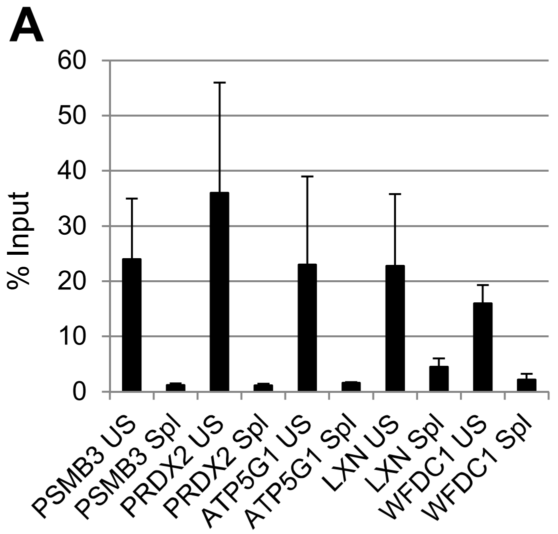


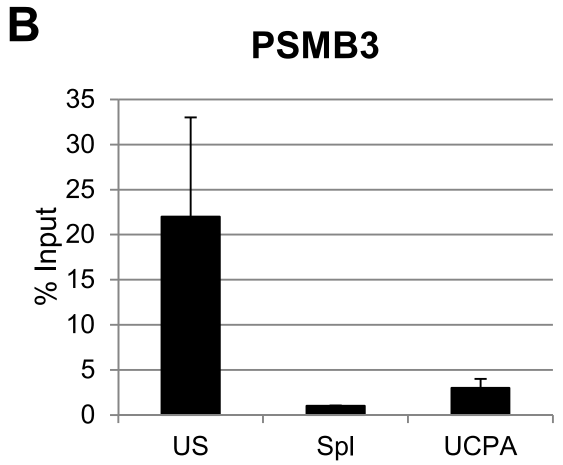


**Supplementary Figure 5**

A

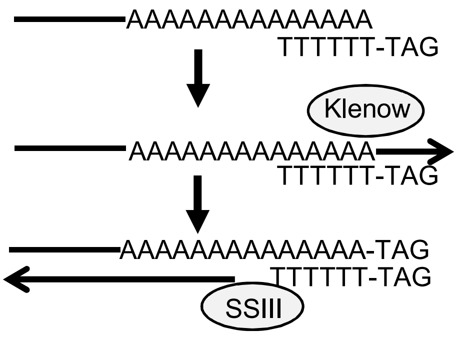


B

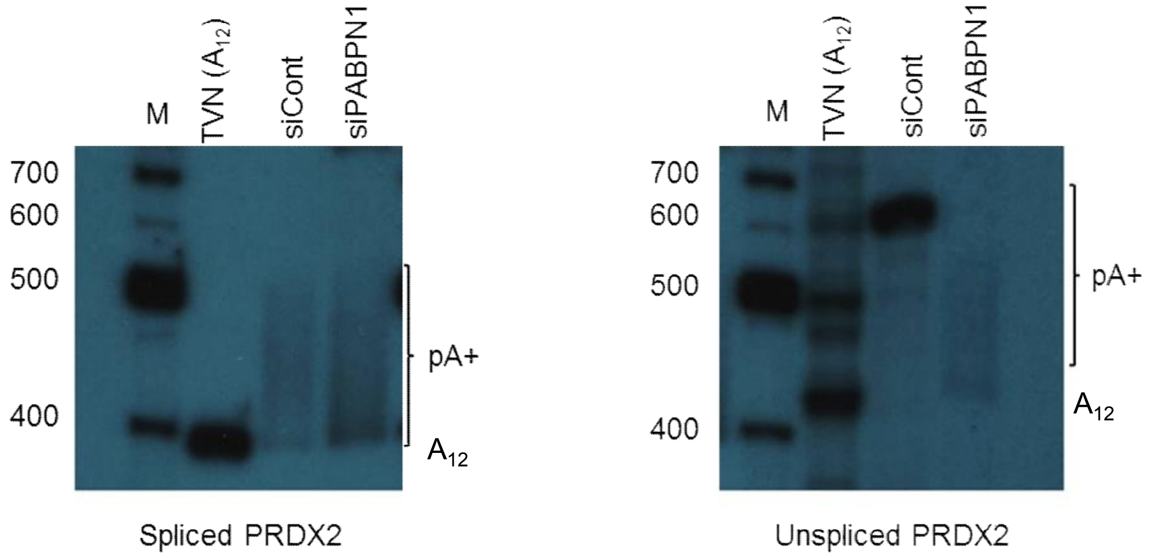


**Supplementary Figure 6**

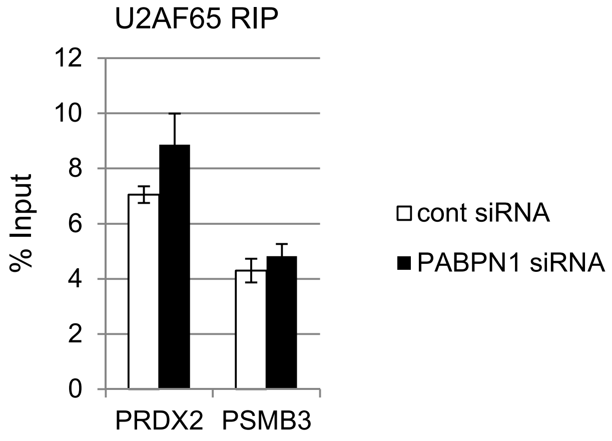


**Supplementary Figure 7**

## SUPPLEMENTARY FIGURE LEGENDS

### Supplementary figure 1

Sequence of the synthesised WT PABPN1 engineered to be RNAi-resistant. 5' and 3' regions of overlap with the cloning vector are also shown.

### Supplementary figure 2

A. Northern blotting analysis of β-globin RNA from βWT and βA46δ cells. Oligo dT was used in conjunction with RNAse H to collapse any polyadenylated species (shown in +dT lanes). This treatment reduced both βWT and βA46δ transcripts to a single sharp band. The blot was probed with a 5' labelled oligo complimentary to β-globin exon 3. Like βWT transcripts, βA46δ transcripts are subject to polyadenylation activity because, in the absence of oligo-dT, a smear of polyadenylated products is observed rather than a sharp band as would be expected if all transcripts terminated at the RZ cleavage site.

B. Cloning and sequencing of poly(A) tails derived from βA46δ transcripts. Two representative sequence traces are shown showing poly(A) tails of 63 and 66 nucleotides. Importantly, there are only 46 templated A tails upstream of the RZ cleavage site and so this experiment provides direct evidence that βA46δ transcripts are further polyadenylated in cells following RZ cleavage. This is consistent with the smeared pattern of polyadenylation observed by Northern Blotting of RNA deriving from this construct.

### Supplementary figure 3

RNA-immunoprecipitation analysis of PABPN1 binding to unspliced and spliced βWT or βA46δ transcripts. Graph shows percentage input after normalising to PSMB3 pre-mRNA levels. Note that a higher percentage of unspliced RNA is bound than spliced RNA. This is

likely to be because many spliced transcripts will be in the cytoplasm where PABPN1 is not present.

**Supplementary figure 4**

Northern blot analysis of β-globin RNA isolated from βWT, βA46δ, βMENβδ and βA60MENβδ transfected cells. RNA was pre-cleaved in exon 3 using an oligo and RNAse H before electrophoresis. The blot was probed with a 5' end labelled oligo complimentary to exon 3. While βWT and βA46δ transcripts are polyadenylated (indicated by smear) both βMENβδ and βA60MENβδ transcripts are not and appear as a sharp band. βA60MENβδ transcripts migrate higher than βMENβδ transcripts due to the internal A-tail. Note that this is the same blot that appears in main text Figure 2C.

**Supplementary figure 5**

**A.** RIP analysis of PABPN1 binding to unspliced and spliced polyadenylated PSMB3, PRDX2, LXN, ATP5G1 and WFDC1 transcripts. Reverse transcription was with oligo-dT. Quantitation is shown as % of input. Note that a higher percentage of unspliced RNA is bound that spliced RNA. This is likely to be because many spliced transcripts will be in the cytoplasm where PABPN1 is not present.

**B.** RIP analysis of PABPN1 binding to spliced, unspliced and non poly(A) site cleaved (UCPA) PSMB3 transcripts. Reverse transcription was with random hexamers. Again, unspliced RNA is bound most efficiently. Note that UCPA transcripts are poorly bound, which confirms that robust PABPN1 binding requires a poly(A) tail.

**Supplementary figure 6**

A. Overview of ePAT assay used to determine poly(A) tail length (1). A tagged oligo-dT primer (TTTTTT-TAG) is annealed to the RNA and the poly(A) tail is then extended with klenow to achieve complementarity with the TAG part of the primer. Reverse transcription is then performed with superscript III (SSIII) at 55 degrees. At this

temperature, primers not extended by klenow (e.g. as a result of binding internally within poly(A) tails) will dissociate from the RNA. Because only oligos that annealed to the very 3' end of the poly(A) tail are extended by klenow, this procedure selects for cDNA synthesis from the very 3' end of the tail.

B. ePAT analysis of the spliced and unspliced human PRDX2 transcripts from cells treated with control or PABPN1 siRNAs. PCR amplicons were resolved on a 5% denaturing polyacrylamide gel. The TVN-PAT reaction represents the size of the amplicon with 12As and derives from a parallel reverse transcription in which cDNA synthesis was from the very 5' end of the poly(A) tail. Most notably, PABPN1 depletion caused a substantial shortening of poly(A) tails on unspliced PRDX2 transcripts. The effect on poly(A) tails at the 3' end of spliced RNA was more modest. Note that because unspliced RNA is less abundant in control cells, more of this cDNA was amplified to aid visualisation.

**Supplementary figure 7**

RNA immunoprecipitation analysis of terminal PRDX2 and PSMB3 introns using the U2AF65 antibody performed in the absence of cross-linking, in control and PABPN1-depleted cells. Quantitation shows % of input. Unlike when UV cross-linking is used prior to immunoprecipitation there is no PABPN1-dependent reduction in binding. Data were normalised to Myc In2-Ex3 levels.

**Supplementary references**

1. **Janicke A, Vancuylenberg J, Boag PR, Traven A, Beilharz TH.** 2012. ePAT: a simple method to tag adenylated RNA to measure poly(A)-tail length and other 3' RACE applications. RNA **18:**1289-1295.

| Primers used for Q-PCR analysis | |
|---|---|
| PSMB3 unspl term int F | 5' ATTTGTTCTGCTCCCTTGCC 3' |
| PSMB3 unspl term int R | 5' CATTCGGGCCTTCAGTGTC 3' |
| PSMB3 spl term int F | 5' TGAAACCATCTCCCAAGCCA 3' |
| PSMB3 spl term int R | 5' CCTGGTGGTGATTTTGTCCT3' |
| PSMB3 cotrans unspl F | 5' CTTGTGATTTTCTCCCTCTGC 3' |
| PSMB3 cotrans unspl R | 5' GGCAGAGATCTGCAGGAAAG 3' |
| PSMB3 cotrans spl F | 5' TGGGAGTCATTGTCCACAT 3' |
| PSMB3 cotrans spl R | 5' GGCAGAGATCTGCAGGAAAG 3' |
| PSMB3 unspl int2 F | 5' AGATGGTGACCACGGACTT 3' |
| PSMB3 unspl int2 R | 5' TTGGATGGTCCAAGAAGAGG 3' |
| PSMB3 spl int2F | 5' CCGGCTGTACATCGGTCT 3' |
| PSMB3 spl int2 R | 5' CACCATGCTCATGAGGGTAT 3' |
| PSMB3 UCPA F | 5' TCACCACCAGGACACTGAAG 3' |
| PSMB3 UCPA R | 5' GGCAGAGATCTGCAGGAAAG 3' |
| PRDX2 unspl term int F | 5' CCCTTGCTTTGCTTCACCTT 3' |
| PRDX2 unspl term int R | 5' CACTATCCGTTAGCCAGCCT 3' |
| PRDX2 spl term int F | 5' CTCCGTGGATGAGGCTCTG 3' |
| PRDX2 spl term int R | 5' GGCTTAATCGTGTCACTGCC 3' |
| PRDX2 cotrans unspl F | 5' CATCCTTGTGTCTTCCCACA 3' |
| PRDX2 cotrans unspl R | 5' ACGAAAGGACAGAGCACACC 3' |
| PRDX2 cotrans spl F | 5' AGACGAGCATGGGGAAGTTT 3' |
| PRDX2 cotrans spl R | 5' ACGAAAGGACAGAGCACACC 3' |
| PRDX2 unspl int1 F | 5' GGGTTGGGAGGACAAAGTGT 3' |
| PRDX2 unspl int1 R | 5' TCCGACAGCTTCACCTCTTT 3' |
| PRDX2 spl int1 F | 5' GCCTTTGCCCACGCAGCTTTC 3' |
| PRDX2 spl int1 R | 5' TCCGACAGCTTCACCTCTTT 3' |
| PRDX2 UCPA F | 5' CCTTCTCATGCCTCCACCTA 3' |
| PRDX2 UCPA R | 5' ACGAAAGGACAGAGCACACC 3' |
| LXN unspl term int F | 5' CCAGATAGGCACCAGATTTAAGT 3' |
| LXN unspl term int R | 5' GTACTTCCTTTGGCAGACGG 3' |
| LXN spl term int F | 5' GGAGATTATTCCCTGGCAAATG 3' |
| LXN spl term int R | 5' CCAGTGAAATTGGCATACACATC 3' |
| LXN unspl int1 F | 5' GCCTTGGTGGCACAGAACTA 3' |
| LXN unspl int1 R | 5' ATCCTGAAGCCTCTGCTGTC 3' |
| LXN spl int1 F | 5' GCCTTGGTGGCACAGAACTA 3' |
| LXN spl int1 R | 5' GGTGATACTTATGTCCTCTTCC 3' |
| ATP5G1 unspl term int F | 5' CCTCAACTGGCAATGATCTCTG 3' |
| ATP5G1 unspl term int R | 5' GCAGTAGCAACAGGCCGGTG 3' |
| ATP5G1 spl term int F | 5' GAACCCGTCTCTCAAGCAGC 3' |
| ATP5G1 spl term int R | 5' GCAGTAGCAACAGGCCGGTG 3' |
| ATP5G1 cotrans unspl F | 5' CCTCAACTGGCAATGATCTCTG 3' |
| ATP5G1 cotrans unspl R | 5' GCTGAGCAAATTCCCTCTCCTC 3' |
| ATP5G1 cotrans spl F | 5' GAACCCGTCTCTCAAGCAGC 3' |
| ATP5G1 cotrans spl R | 5' GCTGAGCAAATTCCCTCTCCTC 3' |
| ATP5G1 unspl int2 F | 5' ACTGATTTGGTAGGATGTGGC 3' |
| ATP5G1 unspl int2 R | 5' TCACTGGGCTATTCAAGAAGGA 3' |

| | |
|---|---|
| ATP5G1 spl int2 F | 5’ CGGGGCATTATTCATTTCTCCA 3’ |
| ATP5G1 spl int2 R | 5’ TGTTTAGATGAATTCACTGGGCT 3’ |
| WFDC1 unspl term int F | 5’ GCGGTTCACCCTGATCCTTCT 3’ |
| WFDC1 unspl term int R | 5’ GGCCTTGCTCCCAGGATGGT 3’ |
| WFDC1 spl term int F | 5’ GGGACAACAGAAGCACTTTCAGT 3’ |
| WFDC1 spl term int R | 5’ GGCCTTGCTCCCAGGATGGT 3’ |
| WFDC1 unspl int1 F | 5’ TTGCTACTTCTCCTCCACGC 3’ |
| WFDC1 unspl int 1 R | 5’ GTGTCTAGGGATCGCCTCTC 3’ |
| WFDC1 spl int1 F | 5’ AGAAATCCCGTGCCGAGG 3’ |
| WFDC1 spl int1 R | 5’ GCATCCGTTGTAGCAGCAG 3’ |
| GAPDH unspliced F | 5’ GGGCCAGAGACTGGCTCTTA 3’ |
| GAPDH unspliced R | 5’ TTCCTCTTGTGCTCTTGCTG 3’ |
| GAPDH spliced F | 5’ CTGACTTCAACAGCGACACC 3’ |
| GAPDH spliced R | 5’ GTGGTCCAGGGGTCTTACTC 3’ |
| Myc unspliced F | 5’ CATGCCTTGTATTTGTACAGCA 3’ |
| Myc unspliced R | 5’ CTCTGACCTTTTGCCAGGAG 3’ |
| Myc spliced F | 5’ CCAGCAGCGACTCTGAGGAG 3’ |
| Myc spliced R | 5’ GCTGTGGCCTCCAGCAGAAG 3’ |
| β -globin unspl int1 F | 5’ ACGTGGATGAAGTTGGTGG 3’ |
| β -globin unspl int1 R | 5’ TGCCTATCAGAAACCCAAGAG 3’ |
| β -globin spl int1 F | 5’ TGAGGCCCTGGGCAGGTTGG 3’ |
| β -globin spl int1 R | 5’ CACTCAGTGTGGCAAAGGTG 3’ |
| β -globin unspl int2 F | 5’ GAGTCCAAGCTAGGCCCTTT 3’ |
| β -globin unspl int2 R | 5’ ACCAGCCACCACTTTCTGAT 3’ |
| β -globin spl int2 F | 5’ AGTGCTCGGTGCCTTTAGTG 3’ |
| β-globin spl int2 R | 5’ GCCACCACTTTCTGATAGGC 3’ |
| **Primers used for cloning** | |
| δRZ F | 5' AGGGCGGCATGGTCCCAGCCTCCTCGCTGGCGCCGCCTGGGCAACATGCT TCGGCATGGCGAATGGGACCAAA 3' |
| δRZ R | 5’TTTGGTCCCATTCGCCATGCCGAAGCATGTTGCCCAGGCGGCGCCAGCGA GGAGGCTGGGACCATGCCGCCCT 3’ |
| β-globin vector F | 5’CCTTGGGAAAATACACTATATCT 3’ |
| β-globin vector R | 5’ATCCAGATGCTCAAGGCCCTTCA 3’ |
| A46 δRZ F | 5’ AAAAAAAAAAAAAAAAAAAAAAAAGGGCGGCATGGTCCCAGCCT 3’ |
| A46 δRZ R | 5’ TTTTTTTTTTTTTTTTTTTTTTTTATCCAGATGCTCAAGGCCCTTCA 3’ |
| MENβ F | 5’ GGTGTTTCTTTTACTGAGTGCAGCCCATGGCCGCACTCAGGTTTTGCTT TTCACCTTCCCATCTGTGAAAGAGTGAGCAGGAAAAAGCAAA 3’ |
| MENβ R | 5’ TTTGCTTTTTCCTGCTCACTCTTTCACAGATGGGAAGGTGAAAAGCAAAA CCTGAGTGCGGCCATGGGCTGCACTCAGTAAAAGAAACACC 3’ |
| His SL F | 5’ TCAGGGCCAGGGCGGCATGGTCCCAGCCTCCT 3’ |
| His SL R | 5’ TAAGGGCCATCCAGATGCTCAAGGCCCTTCA 3’ |
| Internal A20F | 5’ AAAAAAAACTGGGGGATATTATGAAGGGCC 3’ |
| Internal A20 R | 5’ TTTTTTTTTTTTAGTAGTTGGACTTAGGGAAC 3’ |
| Internal A40 F | 5’ AAAAAAAAAAAAAAAAAAACTGGGGGATATTATGAAGGGCC 3’ |
| Internal A40 R | 5’ TTTTTTTTTTTTTTTTTTTTTAGTAGTTGGACTTAGGGAAC 3’ |
| Internal A60 F | 5’ AAAAAAAAAAAAAAAAAAAAAAAAAAAACTGGGGGATATTATGAAGGGCC 3’ |
| Internal A60 R | 5’ TTTTTTTTTTTTTTTTTTTTTTTTTTTTTTTTAGTAGTTGGACTTAGGGAAC 3’ |
| **Primers used for ePAT assay** | |
| ePAT RT | 5’ GCGAGCTCCGCGGCCGCGTTTTTTTTTTTT 3’ |

| | |
|---|---|
| ePAT TVN RT β-globin | 5' GCGAGCTCCGCGGCCGCGTTTTTTTTTTTTGCA 3' |
| ePAT TVN RT PRDX2 | 5' GCGAGCTCCGCGGCCGCGTTTTTTTTTTTTCAC 3' |
| ePAT TAG R | 5' GCGAGCTCCGCGGCCGCG 3' |
| β-globin unspl F | 5' GAGTCCAAGCTAGGCCCTTT 3' |
| β-globin spl F | 5' AGTGCTCGGTGCCTTTAGTG 3' |
| PRDX2 unspl F | 5' CCTTGAACCTGGGGCTGTAG 3' |
| PRDX2 spl F | 5' CAGACGAGCATGGGGAAGTTTG 3' |
| **Primers used for Northern blot** | |
| β-globin ex3 RNase H | 5' TGAATTCTTTGCCAAAGTGATG 3' |
| β-globin ex3 R probe | 5' CTTGTGGGCCAGGGCATTA 3' |
| **siRNAs** | |
| PABPN1 | Life technologies silencer select (s15626) |
| PAPα | Life technologies silencer select (s21458) |
| PAPγ | Life technologies silencer select (s35066) |
| Rrp40 | Life technologies silencer select (s27229) |
| Rrp6 | Target: cauuaaggaucgaaguaaa |

# Supplementary Table 1
